# Corruption in the health care sector: A barrier to access of orthopaedic care and medical devices in Uganda

**DOI:** 10.1186/1472-698X-12-5

**Published:** 2012-05-03

**Authors:** Maryse Bouchard, Jillian C Kohler, James Orbinski, Andrew Howard

**Affiliations:** 1 Leslie Dan Faculty of Pharmacy, University of Toronto, Toronto, ON, Canada; 2 Division of Orthopaedic Surgery, Hospital for Sick Children, University of Toronto, Toronto, ON, Canada; 3 Division of Global Health, Dalla Lana School of Public Health, University of Toronto, Toronto, ON, Canada; 4 Munk School of Global Affairs, University of Toronto, Toronto, ON, Canada; 5 Department of Health Policy, Management and Evaluation, Faculty of Medicine, University of Toronto, Toronto, ON, Canada

## Abstract

**Background:**

Globally, injuries cause approximately as many deaths per year as HIV/AIDS, tuberculosis and malaria combined, and 90% of injury deaths occur in low- and middle- income countries. Given not all injuries kill, the disability burden, particularly from orthopaedic injuries, is much higher but is poorly measured at present. The orthopaedic services and orthopaedic medical devices needed to manage the injury burden are frequently unavailable in these countries. Corruption is known to be a major barrier to access of health care, but its effects on access to orthopaedic services is still unknown.

**Methods:**

A qualitative case study of 45 open-ended interviews was conducted to investigate the access to orthopaedic health services and orthopaedic medical devices in Uganda. Participants included orthopaedic surgeons, related healthcare professionals, industry and government representatives, and patients. Participants’ experiences in accessing orthopaedic medical devices were explored. Thematic analysis was used to analyze and code the transcripts.

**Results:**

Analysis of the interview data identified poor leadership in government and corruption as major barriers to access of orthopaedic care and orthopaedic medical devices. Corruption was perceived to occur at the worker, hospital and government levels in the forms of misappropriation of funds, theft of equipment, resale of drugs and medical devices, fraud and absenteeism. Other barriers elicited included insufficient health infrastructure and human resources, and high costs of orthopaedic equipment and poverty.

**Conclusions:**

This study identified perceived corruption as a significant barrier to access of orthopaedic care and orthopaedic medical devices in Uganda. As the burden of injury continues to grow, the need to combat corruption and ensure access to orthopaedic services is imperative. Anti-corruption strategies such as transparency and accountability measures, codes of conduct, whistleblower protection, and higher wages and benefits for workers could be important and initial steps in improving access orthopaedic care and OMDs, and managing the global injury burden.

## Background

The global injury burden is severely underappreciated by the global health and surgical communities. Orthopaedic injuries and disease account for 14% of the world’s DALYs (Disability Adjusted Life Years) lost and 9% of the world’s mortality according to WHO estimates [1]. DALYs are a measure of the combined impact of the morbidity and mortality of a condition, by adding the years of life lost because of premature mortality with the healthy years of life lost because of disability [2]. The burden of chronic disability is large compared with premature mortality, but remains poorly measured. Ninety percent of injury deaths occur in low- and middle-income countries [3]. Globally, injuries cause approximately as many deaths per year as HIV/AIDS, tuberculosis and malaria combined [4]. By 2020, road traffic crashes are expected to be the leading cause of DALYs worldwide [4]. By the year 2030, road traffic crashes, a major cause of orthopaedic injury, are predicted to be the third leading cause of long-term disability globally [5]. This is an area that has surprisingly not been a global health priority; we hope this paper will draw much needed global attention to this issue.

Timely access to appropriate orthopaedic care can prevent death, and disability due to infections, permanent deformities, chronic pain, or inability to ambulate [6]. As many orthopaedic procedures require an orthopaedic medical device (OMD), such as plaster, an external fixator or an implant [6], accessibility and availability of these devices are essential.

A qualitative case study was conducted in Uganda in November and December 2010, investigating the accessibility of OMDs. Interviews explored participants’ perceptions of the major barriers to availability of OMDs and solutions for improving access. The global governance and global health literature confirm that corruption is a major impediment to access of health care in low-income countries [7]. This paper is the first to explore how corruption specifically hinders access to orthopaedic care and orthopaedic medical devices, and exacerbates the global injury burden.

## Corruption in the health care sector

Corruption, as defined by Transparency International, is the “abuse of entrusted power for private gain” (p.xvii) [8]. With health care expenditures totaling 3 trillion US dollars globally, this sector is particularly vulnerable to corruption [8]. In health care, there is a significant diversity of services offered, and a large scale and expense associated with procurement [9]. In addition, the nature of health care is such that the demand is not fully predictable and often exceeds supply [9]. Health care systems also tend to have weak or non-existent rules and regulations, lack of accountability, information imbalances between providers and patients and suppliers and providers, and low salaries for health care professionals and public officials [7]. These characteristics of the health sector make it susceptible to corruption. Corruption has been well documented in the health sector in Uganda. A study in Uganda reported that the resale of pharmaceuticals is the greatest source of income for health care workers [10]. In Uganda, it is estimated that over two-thirds of drugs meant for free distribution in the public sector were lost due to theft or went unaccounted for, and that 68-77% of formal user charges were misappropriated or pocketed by workers [11]. In 2005, the Global Fund for HIV/AIDS, Malaria and Tuberculosis suspended all donations to Uganda when over 1.6 million US dollars of grants went missing [12]. To date, two Ugandan officials have been accused and sentenced for the embezzlement of Global Fund monies [13].

Theft, diversion and resale of drugs are other sources of corruption documented to occur at the distribution point of pharmaceutical supply chain [9]. For example, there can be theft without falsification of inventory records, dispensing of drugs to patients who did not actually attend the pharmacy or clinic, recording of drugs as dispensed to legitimate patients but the patients do not receive them, and dispensing of drugs to patients who pay for them but the health care provider keeps the funds for themself [14]. Corruption in orthopaedics was an uncommon topic in academic literature and non- medical media until the announcement of a large-scale lawsuit in the United States in 2005. The Department of Justice’s U.S. State Attorney for the District of New Jersey brought forth allegations against the five largest orthopaedic device manufacturers for illegal kickbacks to surgeons and false claims allegations. Physicians were allegedly awarded vacations, gifts and annual “consulting fees” as high as $200,000 in return for physician endorsements of their implants or use of them in operations [15]. The false claims were concerning illegal promotions of off-label uses for a certain product. These five companies control 95% of the orthopaedic medical device market [15]. Four of those companies paid 311 million US dollars to settle the case [15]. Many of the individual orthopaedic surgeons are the subjects of investigations as well.

The medical device industry, especially in orthopaedics, is an area where there are relatively large sums of money involved and thus a susceptibility to corruption [9]. Corruption within orthopaedic services and industry may inflate prices of equipment, lower the quality of care and products, and impede the necessary response to alleviating the growing global injury burden. Previous reports have established that corruption is present in the health care system in Uganda, and in the orthopaedic device system elsewhere. We are aware of no prior work studying corruption in the orthopaedic device system in Uganda, nor in any similar country. This case study conducted in Uganda identifies the areas in which orthopaedic services are susceptible to corruption and how it affects patient care. The data also suggest mechanisms that can be applied as anti- corruption measures.

## Methods

To explore the phenomenon of access to OMDs in low-income countries, a descriptive qualitative case study was undertaken in Uganda in November and December 2010. Open-ended interviews were used to elicit the experiences of key informants in accessing OMDs. Given the limited literature on the subject, this approach was chosen given it is useful for extracting information and investigating a phenomenon for which little is known [16]. Importantly, case studies are subject to less bias than other qualitative methodologies, as preconceived theories are not easily imposed on the data [17]. Uganda was selected for the case study as it has a similar health care infrastructure to many other low-income countries, and experiences many of the economic constraints common to such settings [18].

## Sampling

To collect the most information-rich data, purposive sampling and snowball sampling were employed. Four categories of participants were pre-selected: 1) surgeons who perform orthopaedic procedures and other related healthcare professionals; 2) hospital administrators and government officials responsible for procuring OMDs; 3) orthopaedic patients; and 4) orthopaedic industry representatives. Participants were chosen to ensure the sample represented professionals and patients from urban and rural areas, academic and district hospitals, and public and private hospitals. The primary unit of sampling was the health centre. There are only 28 orthopaedic surgeons in Uganda [19] and they are mostly concentrated in the capital city, but are also present in some district, mission, non- governmental and private hospitals in rural areas of the country. Within each health centre, we attempted to identify and interview representatives of each category of participant, if present. A small number of interviews were conducted at the international meeting of the College of Surgeons of Central, Eastern and Southern Africa (COSECSA). Given that not all participants could be identified prior to commencing the study in Uganda, snowball sampling was required. The sample size was determined using the saturation principle [20]. Although snowball sampling may lead to bias by people with similar views being more likely to know each other, we believe that interviewing more than half the orthopaedic surgeons in the country severely restricted this form of bias.

## Data collection

Data collection was achieved through open-ended semi-structured interviews conducted by the first author. For example, the first question for each interview broadly asked participants to describe their experience in accessing orthopaedic medical devices in their hospital. All interviews were recorded with hand-written notes and transcribed by the same author. Although the original intent had been to digitally audio record the interviews, the interview settings were often noisy and informants were more forthcoming with information when the digital audio recorder was off. Note-taking therefore became the preferred method of recording. This is in keeping with experiences in other studies [21]. To contextualise the interview data, the first author participated in clinical activities and kept a journal as observational field notes.

Written and oral informed consent was obtained from all participants prior to their participation in the interview process. Research ethics approval was obtained from the Institutional Review Boards at the University of Toronto, the home institution of the authors, and Makerere University in Kampala, Uganda. This study was conducted in compliance with the guidelines of the Helsinki Declaration.

## Data analysis

The qualitative method of thematic analysis was used to analyze the data. This method identifies, analyses and describes patterns and themes within data [22]. Nvivo 9 Software was used to create a database to organize, code and categorize the collected interview data. According to the methodology described by Fereday and Muir-Cochrane [23], the first step of coding was to review the data. This involved transcribing, re-reading and, summarizing each interview, and tabulating the participants’ demographics. The second step of coding, and the third step of grouping codes into themes, were performed simultaneously. Finally, themes were condensed into over-arching categories that represented and accurately explained the data set.

## Results

Forty-five interviews were conducted in 12 healthcare centers across Uganda. Table 1 summarizes the study sample demographics. Participants included 16 orthopaedic surgeons, 13 health care professionals who were not orthopaedic surgeons, 8 industry representatives, 6 patients, and 2 government officials. Fifty-one percent of participants worked in or attended only public institutions, 54% of the healthcare professionals worked in academic centers, and 49% had a part-time or full-time private practice. Seven of the participants practiced surgery in African countries other than Uganda. These interviews were conducted to include regional context, specifically to find out whether Uganda was unique, or was similar to its neighbors. The purpose was not to study Africa in general, nor to extend the present results beyond Uganda. Few government officials were available for interview. This is not surprising given the sensitive nature of the topic.

**Table 1:** Study sample participants

**Sample sub-group**	**Number of participants (% of Total sample)**
Health care workers	29 (65%)
Orthopedic surgeons	16 (36%)
Other health care workers	13 (29%)
Health care administrators	2 (4%)
Government officials	2 (4%)
Hospital administrators	0 (0%)
Patients	6 (13%)
Industry representatives	8 (18%)
Public practice on	18 (51%)
Private practice only	3 (9%)
Public and private practice	14 (40%)
Academic affiliation or institution	19 (54%)
No academic affiliation	16 (46%)
Urban practice location	24 (53%)
Rural Practice location	21 (47%)
Practice in Uganda	38 (84%)
Practice in other Africa country*	7 (16%)

*Ethiopia, Kenya, Malawi, Rwanda, and South Africa.

**Figure 1:**
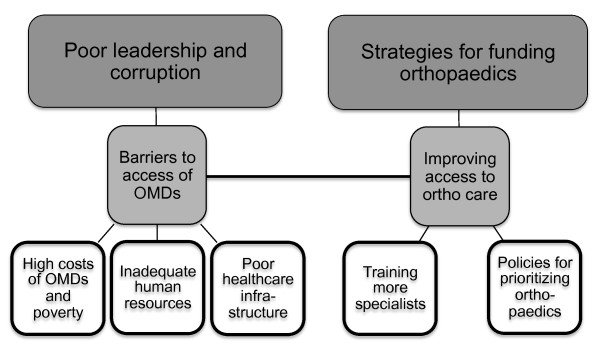
Overarching categories from interview data.

Data analysis produced hundreds of codes and dozens of themes. Dominant and important themes stated by the majority of participants can be found in Table 2. Codes and themes were condensed and revealed two overarching categories, as seen in Figure 1: Barriers to access of orthopaedic services and OMDs, and mechanisms for improving access to orthopaedic care. Of the four major barriers, poor leadership and corruption was the most unexpected but significant finding. The three other themes were high costs of OMDs and poverty, inadequate human resources and training, and inefficient and insufficient healthcare infrastructure. The second category on improving access to orthopaedic care identified policies for prioritizing orthopaedic services, training of more orthopaedic specialists, and the adoption of strategies for raising funds for orthopaedics. The discussion of this paper focuses on the theme of corruption and explores the participant-elicited policies and funding strategies that could serve as anti- corruption mechanisms.

**Table 2:** Sample of preliminary codes and themes from interview data

**Line-by-line codes**	**Number of references in coded text**
Cost of OMDs	32
Local manufacturing	32
Patient or hospital buys from distributor	26
Government supply	24
Donations	23
Government budget	21
Poverty	21
Availability and Accessibillty of OMDs	19
Lack of heath infrastructure	17
Procurement	17
Policies to prioritize ortho and trauma	14
Reallocation of health care dollars	13
Need more specialists	12
Policies and law enforcement	12

## Discussion

### Barriers to orthopaedic care and medical devices

One of the largest identified obstacles in providing appropriate care to injured patients was perceived corruption. Participants easily identified many of the corrupt practices common to the health care sector at worker, hospital and government levels. These included misappropriation of funds, theft of equipment, resale of drugs and medical devices, fraud and absenteeism. They expressed the need for anti-corruption measures and improved transparency in government.

### Misappropriation of funds

Participants reported theft and misuse of resources both financial and material. Both government funds and grants from donor agencies were reported as lost or misappropriated.

“Money in the government is not always accounted for and doesn’t always make it to the hospitals.” [Health care professional]

“There is a lot of corruption and wasted money in the government. For example, here there is a 60 000 US dollar Toyota Prado for the medical superintendent to use. It sits in the front of the hospital. It was bought by the government. I see the superintendent use it maybe only once or twice a day” [Health care professional]

“Why can people afford to buy 4x4s but not pay for their health care? In the end, I think that all East African countries can afford [orthopaedic] implants if they use their public money properly. There is so much wasted money.” [Industry representative]

Participants also identified a lack of transparency and accountability in government policy decisions (particularly for health budget allocation) and procedures in the health system. A dominant theme identified was the lack of publicly disclosed oversight of government procurement procedures. Donated funds are typically associated with a higher degree of oversight and accountability, and one participant suggested that donated funds for health care might encourage more transparency.

### Theft and resale of drugs and medical equipment

In Uganda’s universal healthcare system, the government attempts to supply all its hospitals with the necessary equipment and medicines. Hospitals can access what they need through the National Medical Stores, a centralized depot of medical supplies run and paid for by the government [24]. The National Medical Stores however, do not regularly carry orthopaedic equipment other than plaster, forcing orthopaedic departments to rely on donated supplies. Many other supplies and drugs are also frequently out of stock or cost more than hospitals or patients can afford.

“[There is] corruption. The health care budget has a budget for sutures, but when we ask for them they never come.” [Health care professional]

Depleted stocks and high costs force hospitals, physicians and patients to buy from private pharmacies or distributors (Observational Field Notes). This can also incite theft. Stolen orthopaedic implants, donated or government supplied, are reported taken from public hospitals and resold to patients in private hospitals (Observational Field Notes). The lack of inventory systems, clear policies, price lists, and oversight mechanisms make the health care system vulnerable to corrupt practices.

“There is theft of equipment in government hospitals by people who want to set up their own practice. Some stolen instruments are consumables but some are permanent fixtures.” [Health care professional]

Interviews with orthopaedic industry representatives and orthopaedic surgeons who work in other low-income countries reveal the problems of theft and resale also exist outside Uganda. These participants relate the problems of theft to the lack of suppliers of medical equipment, making medical devices difficult to come by even in legitimate ways.

“Once in the 1970’s, Synthes [one of the big five orthopaedic medical device manufacturers] set up a plant in India. Everything they made in India was sent directly to Switzerland, so it could not be sold directly to the market. The labor was really cheap but the quality was the same. But they closed the plant because the manager in India started selling directly to the market. I have tried to convince them to set up a plant in Kenya so Africans can have better access to implants, but they say ‘we don’t want to start a plant here’. They fear their products will end up on the black market.” [Industry representative]

### Fraud

Uganda, like most low-income countries, has a serious lack of orthopaedic specialists, from surgeons to orthopaedic officers and nurses [19]. There are only 28 orthopaedic surgeons in Uganda [19], and only a handful in rural areas [25]. There is no provision to encourage healthcare professionals to work in underserviced areas; this is not uncommon for many health professions. The government will not fund orthopaedic care or supply orthopaedic equipment to a hospital if there is no orthopaedic surgeon present, compounding this problem. Health care professionals and government officials in many low-income countries are paid meager wages and are rarely rewarded for exceptional performance [26]. The median surgeon salary in a public hospital setting is $6000USD per year, while it is $18000 per year in a private setting [25]. In contrast, the median annual income for a ward nurse is $1000 [25]. Under these conditions, many workers engage in other economic activities during working hours or pursue opportunities for financial gain through public service, including the acceptance of informal payments [9]. One study participant described that in Ethiopia, even private hospitals practice corrupt behaviors in order to make private gains. In this case, it is by overcharging patients for medical equipment.

“The private hospital will charge a patient the same whether they use an Indian [generic] or European [brand name] implant. Unless a patient specifies which one they want, the hospital will use the cheapest device, charge for the most expensive, and take the extra money.” [Health care professional]

In Uganda, as hospital stocks are often depleted, patients are frequently asked to buy drugs or equipment from private distributors. Patients worry however, whether they are getting the best price for the medical equipment and drugs they need. The low wages, lack of benefits, and poorly supplied hospitals of the public health care system motivate surgeons to work in the private system partially or exclusively. Fiftyeight percent of all medical professionals interviewed in Uganda worked at least parttime in the private system. Many participants reported needing the income from the private system to support their families (Observational Field Notes). Even though we focus on corruption in this paper, it is important to note that fraud in the forms of informal payments and absenteeism are often viewed as coping mechanisms for health professionals given their low salaries in the public sector.

The current private system negatively affects the public system. The first author participated in clinical care and in the orthopedic operating room while in Uganda. In the larger cities, the operating room rarely started before 11 AM as the nurse, anesthetist or surgeon was coming from an earlier case at a private hospital (Observational Field Notes). Other times, delays were simply because of lack of motivation from the staff. Since they were salaried it did not matter to them financially how many cases per day they achieved (Observational Field Notes). The growing list of patients waiting for surgery did not seem to inspire the staff to work harder or longer. These findings are consistent with other studies on absenteeism and health worker motivation [7]. Furthermore, there is a significant increased cost to the government and public health sector when such absenteeism occurs. Although meager, the government-paid salaries for full-time health care professionals ultimately pay for only part-time employees when workers spend up to half of their time in a private hospital instead providing undivided care in the public sector. The resulting backlog of patients waiting for care and occupying hospital beds for longer periods also increase health care costs and potentially negatively impact patient outcomes.

We found that another consequence of absenteeism is a slowdown in the procurement system for medical devices. All procurement in Uganda is through a lengthy government process (Observational Field Notes,). Absenteeism of physicians and government officials on the procurement committees further slows the process.

“The supply procurement process is incredible. To prevent corruption, there are two to three committees at the district and hospital levels you have to submit your request to. The process takes 6 to 8 months. It always has to go through the government. It can happen that the committees don’t meet because people are away. This can create yearlong delays. When you finally get the tender, sometimes the supplier in the end is not able to supply, then you have to start all over again.” [Health care professional]

### Law enforcement

Although corruption in law enforcement is not directly related to corrupt behavior in the health sector, most cases of orthopaedic trauma are due to road traffic crashes and lack of regulation on the road. There is little consequence for reckless or unsafe driving because the police rarely halt or prevent it, or alternatively because there is a lack of policy or legislation against a particular act of dangerous driving.

“Law enforcement here is not good. No traffic rules are enforced. Careless driving is performed by everyone here, rich or poor.” [Health care professional]

For example, boda-bodas are the motorcycle taxis in Uganda. A law exists that mandates drivers to wear helmets. Few drivers where helmets, and yet the police rarely discipline the drivers (Observational Field Notes).

Participants hypothesized that similar to health care, police are poorly paid and inadequately rewarded for good performance, they may be unmotivated to properly perform their jobs and more likely to accept bribes or other forms of personal gain. This is also a result of a lack of monitoring and enforcement of professional standards.

“We need increased police enforcement, but I don’t know how to increase their interest. Maybe the police aren’t supervised well, they don’t have enough money or high enough salaries, and they don’t have enough knowledge.” [Government Official]

Many participants related corruption in law enforcement as a barrier to access of orthopaedic care as the unpunished reckless driving consistently causes the disabling and fatal injuries that overload the emergency rooms, wards and operating rooms.

## Adapting Uganda’s health care system to respond to the injury burden by preventing corruption

There is no one-size-fits-all solution to eradicating corruption [7]. A society is less vulnerable to corruption when there is rule of law, transparency, trust, and strong accountability mechanisms [8]. Anti-corruption measures must be tailored to each community and country’s needs, political context, and health care system [8].

Preventative measures against corruption can include procurement guidelines, inspection for quality of medical technologies and drugs, codes of conduct for individuals, institutions and industries, and transparency and monitoring procedures [8].

Study participants called for policies that would deter corrupt behaviors.

“We need to lobby the government to change the attitude towards theft [in the health care setting]. And if we can exclude theft [in the hospitals], we can then tell the government with certainty we used all [the supplies] we have, that it wasn’t enough, and we need more for legitimate reasons and not because staff is stealing.” [Health care professional]

Klitgaard [27] describes three phases in overcoming corruption. First, raising awareness through education of the public, and of decision and policy makers. Second, conducting analyses of points most vulnerable to corruption. Last, determining what prevention strategies would be most effective. To prevent corruption that hinders access to orthopaedic care and medical devices, several mechanisms might need to be developed.

### Reducing incentives of corrupt practices by health care workers

Corrupt behaviors such as absenteeism, theft, fraud, and acceptance of bribes and informal payments have been shown to decrease when health care professionals are paid decent wages proportional to their education, skills and training [8]. Governments can also reduce the occurrence of corruption by monitoring payment schemes, adopting codes of conduct [8], increasing the role of community committees and public scrutiny, and performing analyses of and providing report cards for public services are suggested mechanisms [9].

### Anti-corruption strategies in the pharmaceutical and medical device industries

The corruption literature identifies several strategies for preventing corrupt behaviors in the pharmaceutical sector. These could also be applied to the medical device industry and include:

1) Criteria for selection and pricing based on global standards (eg WHO) [28]

2) Professional and public scrutiny of device selection committees [9]

3) Transparent, written, explicit procurement criteria [28]

4) Media publication of tender results [28]

5) Industry codes of conduct [7]

6) Licensing and inspection of pharmaceutical and medical device suppliers [28]

Of particular concern in Uganda is that all medical devices and drugs for the public health system must be procured and distributed through the government’s National Medical Stores. This system is vulnerable to corruption. The government is the sole regulator. If improperly used or controlled this could enable corruption, whereas if properly used and managed it can discourage corruption. Clarification of chains of authority and accountability processes should be encouraged to ensure ethical business practices [9].

### Strategies for improving transparency

The literature on corruption in health care and participants of the Ugandan case study both call for effective mechanisms for improving accountability and transparency in the health sector. The 2006 Global Corruption Report from Transparency International suggested several immediate strategies [8]. Transparency could be improved with regular publishing of information on health budgets and performance at the national, local and health delivery center levels; independent audits of health funds, coordination of international donor support to the health sector with regular evaluations of their programs in terms of health outcomes, and not level or speed of disbursement. There could also be mechanisms for implementing public oversight of government policies, practices and expenditures. Improved transparency and accountability will ideally discourage embezzlement, theft of medical equipment, and fraud and bribery among government officials, law enforcement officers and health care workers.

### Strategies for creating and implementing codes of conduct

Codes of conduct for health care workers, government officials and industry are required to define appropriate practice in the health care sector, and to deter or punish corrupt behaviour. Codes of conduct should be implemented and promoted through continued training and enforcement [8]. These codes should make explicit reference to preventing corruption and conflicts of interest [8]. Codes of conduct will be meaningless however, if health care workers are not being paid a fair living wage for their work [28].

### Whistleblower protection

Governments should introduce whistleblower protection for individuals in procurement bodies, health authorities, health service providers and suppliers of medicines and equipment [8]. Pharmaceutical and medical device companies should do the same. Corrupt practices by individuals, government or industry should be punished, and where relevant these actors should be excluded from participation in future tendering processes with the government [8]. Conversely, good behavior should be rewarded and publicized [7].

### Reducing incentives for corruption through alternative funding of the health care budget

With a limited health care budget, hospitals and the governments are susceptible to corruption. The participants’ suggestions of innovative strategies for funding orthopaedic care may provide new opportunities for financing health care that promote better transparency and accountability.

“We could put a levy on fuel and vehicles, as drivers and riders are those most involved in road traffic crashes. This money would go directly into orthopaedics for implants and other [OMDs], for salaries to increase motivation and increase the working hours, and for improved call coverage to sustain a 24hour operating theatre.” [Health care professional]

“We can use profits from private surgeries in government hospitals to raise funds for public care. Public hospitals can generate their own money through a private wing.” [Health care professional]

These innovative funding strategies may not in themselves prevent corruption, but if coupled with improved accountability and transparency, significant progress can be made.

## Conclusions

Our qualitative case study of orthopaedic services in Uganda has shown that one of the largest barriers to access of much needed orthopaedic and trauma care is corruption. This was the most unexpected but significant finding of the qualitative study. Corruption in the health care sector has been repeatedly shown to hinder access to needed health services. This is no exception for orthopaedic care. Corruption occurs at the worker, hospital and government levels in the forms of misappropriation of funds, theft of equipment, resale of drugs and medical devices, and fraud. Orthopaedic care is also affected by corruption in law enforcement. As the global burden of injury continues to grow, the need to combat corruption and ensure access to orthopaedic services and medical devices increases. Anti- corruption strategies such as improving transparency and accountability measures in policy, procurement, and payment schemes, codes of conduct, whistleblower protection, and higher wages and benefits for workers could be the first step in improving access orthopaedic care and managing the global injury burden.

## Competing interests

The authors declare that they have no competing interests.

## Authors’ contributions

MB conceived, designed and coordinated the study, and drafted the manuscript. JCK, JO, and AH equally contributed to advising MB on study design and conduction, and in providing revisions to the manuscript. All authors read and approved the final manuscript.

## Pre-publication history

The pre-publication history for this paper can be accessed here:

http://www.biomedcentral.com/1472-698X/12/5/prepub
